# Effects of Trehalose on Thermodynamic Properties of Alpha-synuclein Revealed through Synchrotron Radiation Circular Dichroism

**DOI:** 10.3390/biom5020724

**Published:** 2015-05-04

**Authors:** Paolo Ruzza, Rohanah Hussain, Barbara Biondi, Andrea Calderan, Isabella Tessari, Luigi Bubacco, Giuliano Siligardi

**Affiliations:** 1 Institute of Biomolecular Chemistry of CNR, Padua Unit, Padua 35131, Italy; E-Mails: barbara.biondi@cnr.it (B.B.); andrea.calderan@icb.cnr.it (A.C.); 2 Diamond Light Source Ltd., Harwell Innovation Campus, Chilton, Didcot, Oxfordshire OX11 0QX, UK; E-Mails: rohanah.hussain@diamond.ac.uk (R.H.); giuliano.siligardi@diamond.ac.uk (G.S.); 3 Department of Biology, University of Padua, Padua 35122, Italy; E-Mails: isabella.tessari@unipd.it (I.T.); luigi.bubacco@unipd.it (L.B.)

**Keywords:** trehalose, α-synuclein, synchrotron radiation circular dichroism (SRCD) spectroscopy, osmolytes

## Abstract

Many neurodegenerative diseases, including Huntington’s, Alzheimer’s and Parkinson’s diseases, are characterized by protein misfolding and aggregation. The capability of trehalose to interfere with protein misfolding and aggregation has been recently evaluated by several research groups. In the present work, we studied, by means of synchrotron radiation circular dichroism (SRCD) spectroscopy, the dose-effect of trehalose on α-synuclein conformation and/or stability to probe the capability of this osmolyte to interfere with α-synuclein’s aggregation. Our study indicated that a low trehalose concentration stabilized α-synuclein folding much better than at high concentration by blocking *in vitro* α-synuclein’s polymerisation. These results suggested that trehalose could be associated with other drugs leading to a new approach for treating Parkinson’s and other brain-related diseases.

## 1. Introduction

**Figure 1:**
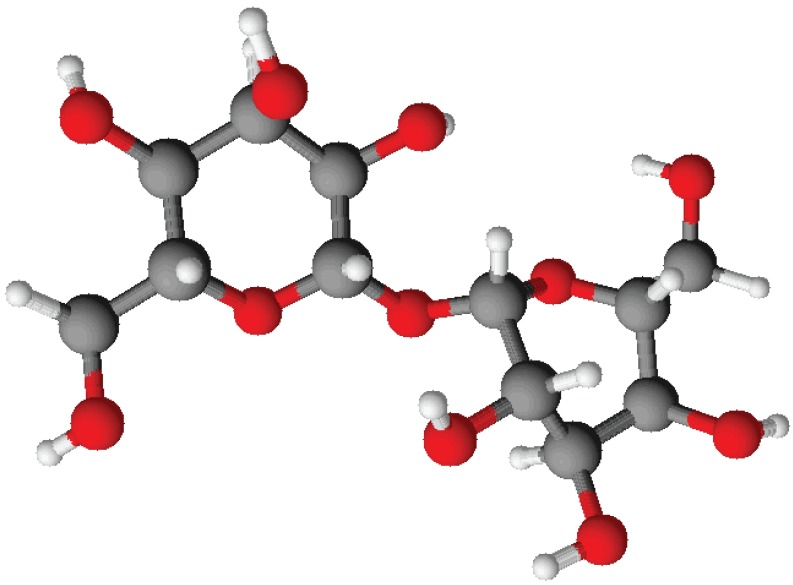
Chemical structure of trehalose.

Trehalose is a non-reducing glucose disaccharide that acts as an osmolyte protecting cells against various environmental conditions and preventing proteins denaturation [1]. Protein misfolding and aggregation are common pathological hallmarks in many neurodegenerative diseases, including Huntington’s, Alzheimer’s and Parkinson’s diseases [2]. The extracellular or intracellular aggregates characterizing these pathologies consist of extended, β-sheet-rich fibril structures that share several biochemical/biophysical properties. Although there is no apparent correlation between the size and/or the primary amino acid sequence of amyloid forming proteins, the common structural motif of protein deposits suggests a conserved mechanism of aggregation pathways [3]. The capability of trehalose (Figure 1) to interfere with protein misfolding and aggregation has been evaluated by several research groups. Tanaka *et al.* [4] demonstrated the potential of trehalose for treating Huntington’s disease, a neurodegenerative disorder characterized by poly-glutamine gain-of-function, showing that this disaccharide is a good inhibitor of protein aggregation. Liu *et al.* [5] found that trehalose *in vitro* both inhibits the aggregation of Aβ40 and Aβ42 peptides and dissolves their preformed aggregates in a dose-dependent mode. At low concentration (<50 mM), trehalose completely inhibits the aggregation of Aβ40 and significantly dissolves its preformed aggregates, while only partially inhibits the aggregation of the more toxic Aβ42 peptide. Moreover, preformed aggregates of Aβ40 co-incubated with 50 mM trehalose were not toxic to human neuroblastoma SH-SY5Y cells.

Recently, trehalose has been tested as inhibitor of α-synuclein aggregation by the Zhung research group [6,7] revealing that low trehalose concentration (10 mM) disaggregated preformed mutated A53T α-synuclein protofibrils and fibrils into smaller aggregates or dissolved into disordered structures. At higher trehalose concentration, up to 100 mM, the transition of A53T α-synuclein into β-sheet structure was slowed down and the formation of mature fibrils completely prevented. It was observed that α-synuclein co-incubated with trehalose assembled into large amorphous aggregates rather than neurotoxic fibrils that after long time incubation with trehalose were re-dissolved into disordered structures. In addition, a lower than 1.0 mM concentration of trehalose was found to inhibit the over-expression of wild-type α-synuclein in transduced PC12 cells protecting the cells against the neurotoxicity induced by α-synuclein [7].

Different hypotheses have been developed to explain the mechanism by which trehalose might stabilize protein folding. It is conceivable that the ability of trehalose -OH groups to form hydrogen bonds with α-synuclein either directly, via the N and O atoms of the amino acid residues, or indirectly, via water molecules hydration shell, may induce the formation of α-synuclein-water-trehalose copolymers [8,9] that prevent the protofibrils formation. Additionally, trehalose was found to be able to protect cells inducing autophagy, a process where cytoplasmic proteins and organelles are sequestered into autophagosomes and delivered to the lysosomes for degradation [10], enhancing the clearance of mutant proteins associated with different neurodegenerative diseases [11,12].

α-Synuclein is a 140-amino acid intrinsically disordered protein involved in different diseases, known as synucleinopathies, including the Parkinson’s disease. In pathological conditions, α-synuclein forms insoluble fibrils and aggregates, known as Lewy’s bodies, in the dopaminergic neurons. The primary structure of α-synuclein is characterized by the presence of seven imperfect repeats of the KTKEGV sequence at the N-terminal, a middle hydrophobic region and a C-terminal region rich in acidic amino acids [13]. The *in vitro* mechanism of α-synuclein aggregation has been studied by different researchers [14] (reference therein), revealing that the hydrophobic region and the negative charged C-terminal region play a key role in forming fibril structures. In addition to protein mutation, different conditions such as the presence of metal ions, pesticides, and organic solvents significantly enhanced the α-synuclein fibrils formation [15,16,17]. In contrast, conditions favoring either more folded conformations or the fully unfolded form have been found to slow or inhibit fibrils formation [18,19]. However, the *in vivo* mechanism of α-synuclein aggregation in the Parkinson’s disease and in other synucleinopathies is still unclear.

In this paper, we present the use of synchrotron radiation circular dichroism (SRCD) spectroscopy to evaluate the dose-effect of the trehalose on the α-synuclein conformation in order to probe the osmolyte’s ability to interfere with the aggregation of α-synuclein.

## 2. Results and Discussion

In our previous works [20,21], we have demonstrated that SRCD spectroscopy is a useful tool to investigate the biochemical key events involved in the formation of protein aggregates. In particular, it has been used for assessing the conformational transition of amyloid proteins and to gain insight both into the interaction of various small molecules with native proteins and into the effects on amyloid aggregation.

Osmolytes occur at very high concentrations in cells (0.1–1 M), they do not bind to proteins, and they have a unique ability to stabilize native folded proteins [22]. The mechanism proposed to explain the effect of osmolytes to stabilize proteins from denaturation was based on an exclusion phenomenon: osmolytes stabilize the native state of proteins because of the dramatic destabilization of the unfolded state [23]. Indeed, they have an unfavorable interaction with the surface of a native protein that increases the free energy of the native state in respect to the free energy of the native state in the absence of osmolyte. Thus, osmolytes stabilize the globular structure of proteins by favoring compaction. Consequently, the addition of osmolytes, in the case of intrinsically disordered proteins may be a disadvantage as compaction can promote protein aggregation [23].

**Figure 2:**
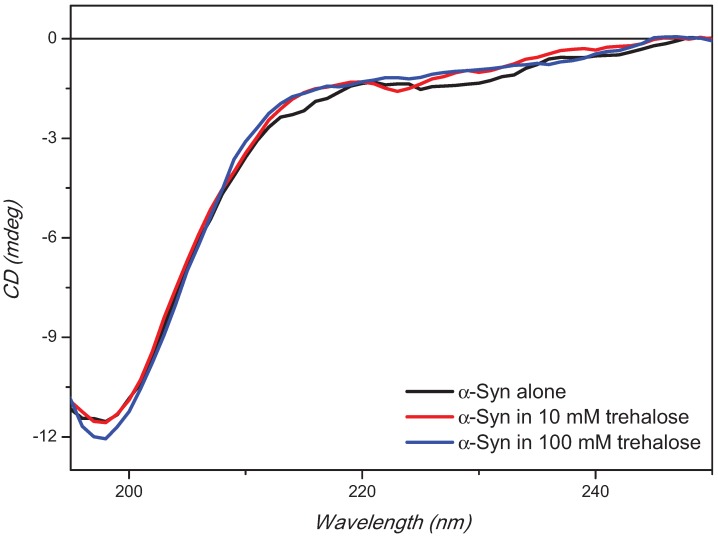
Far-UV synchrotron radiation circular dichroism (SRCD) spectra of α-synuclein alone or in presence of 10 or 100 mM trehalose. α-Synuclein concentration was 0.36 mg/mL in PBS buffer, pH 7.4.

The CD spectrum of α-synuclein showed a negative CD band at about 197 nm characteristic of the disordered structure in aqueous environment (Figure 2). The CD intensity at 197 nm was not affected by the presence of trehalose at low concentration (10 mM), but marginally at higher concentration of 100 mM (Figure 2).

**Figure 3:**
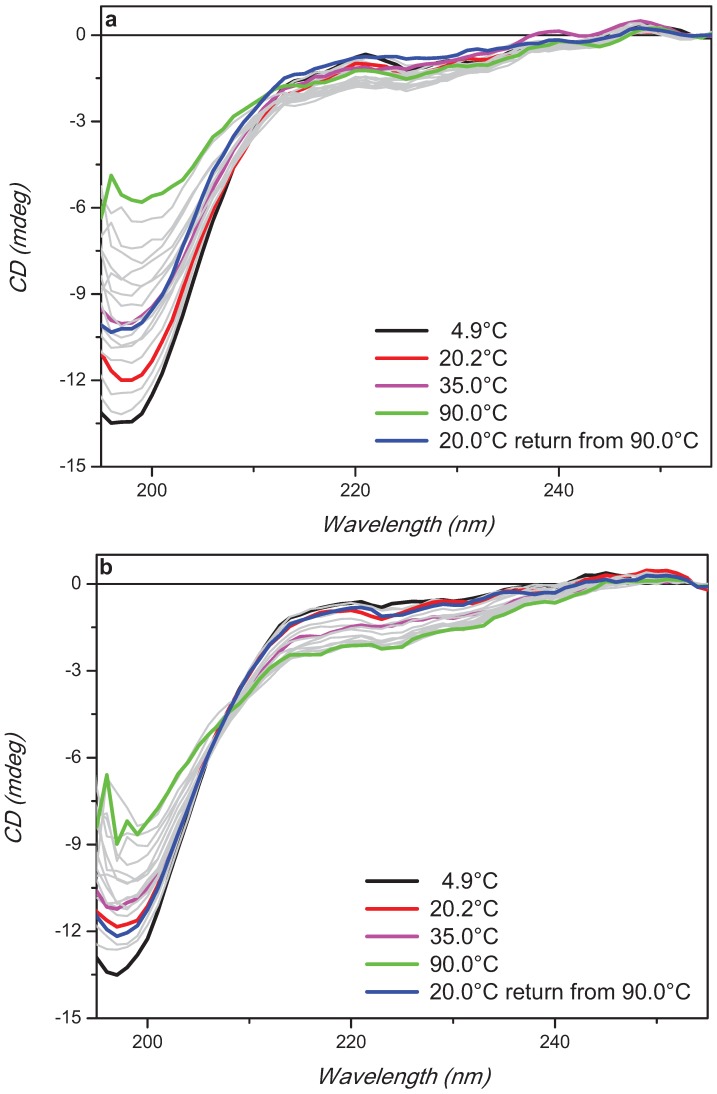

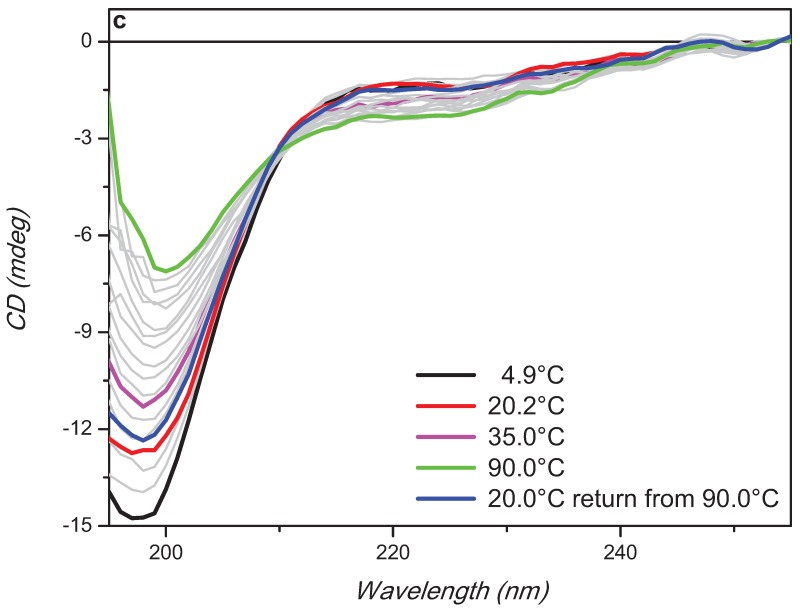
Far-UV SRCD spectra of α-synuclein alone (**a**) or in presence of 10 mM (**b**) or 100 mM (**c**) trehalose at different temperature (indicated). α-Synuclein concentration was 0.36 mg/mL in PBS buffer, pH 7.4.

**Figure 4:**
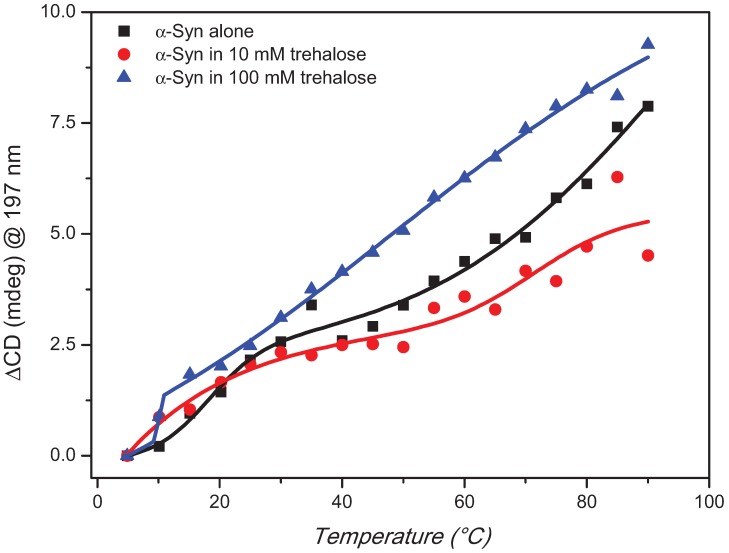
CD melting curves of α-synuclein alone or in presence of 10 or 100 mM trehalose using biDoseResp curve fitting from OriginLab software with R^2^ of 0.98, 0.89 and 0.99 respectively.

The trehalose concentration effect on the stability of aqueous α-synuclein in its disordered native state was assessed by thermal denaturation experiments (Figure 3). Qualitatively the observed CD changes as a function of temperature from 5 °C to 90 °C at 5 °C intervals were similar with a decreased intensity of the negative band at about 197 nm and an increased intensity of the negative shoulder at about 220 nm. Quantitatively, however, the rate of changes were significantly different showing a reduced rate with 10 mM trehalose than that of α-synuclein but an increased one with 100 mM trehalose (Figure 4). The melting process was reversible with 10 mM and almost for 100 mM trehalose but not for α-synuclein (Figure 3) highlighting the different thermodynamic properties induced by trehalose to the protein.

In Figure 4, the thermal changes of α-synuclein with and without 10 mM trehalose were fitted with the biDoseResp equation (using OriginLab software), indicating the presence of two distinct equilibria at temperature either lower or higher than the physiological value (range 30–40 °C). At low temperature, the data being superimposable indicated that the first equilibrium was not affected by the presence of the low concentration of trehalose (10 mM). By increasing the temperature, however, the second equilibrium appeared to be affected by the addition of the osmolyte showing a less steep slope. Such a slope is associated to the heat capacity change (ΔCp) that can be seen as a measure of the exposure of the protein surface to the solvent [24]. Increasing the trehalose concentration to 100 mM, a bigger difference was observed with a steeper straight slope (without double equilibria) than those of α-synuclein with and without 10 mM trehalose.

**Figure 5:**
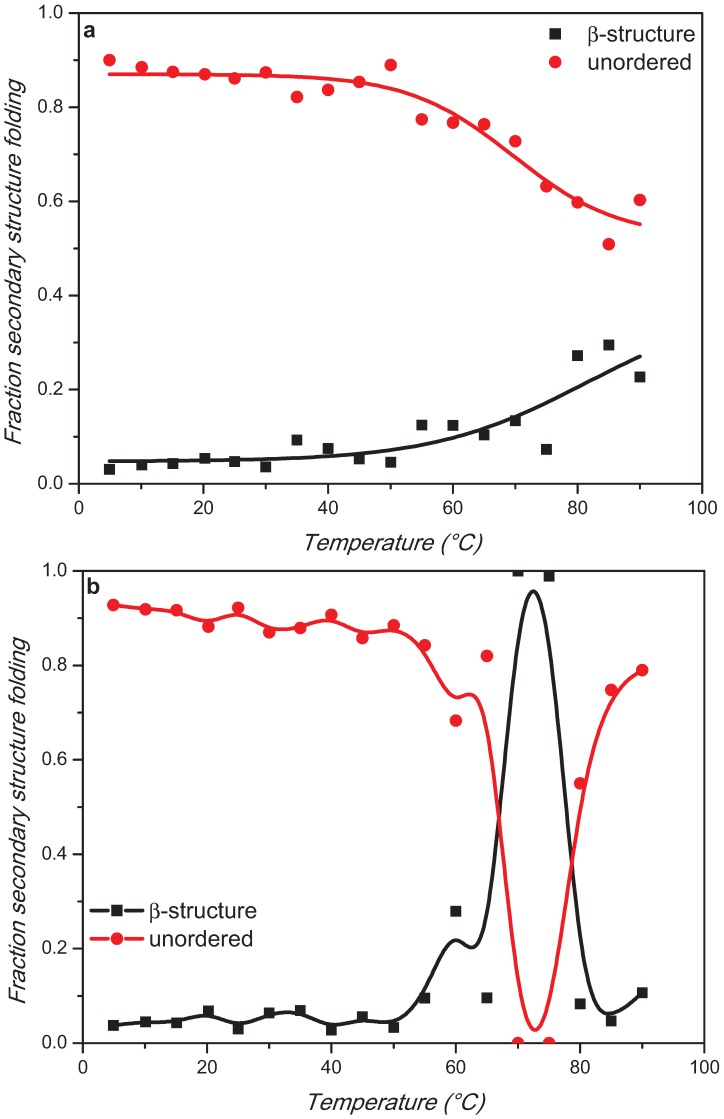

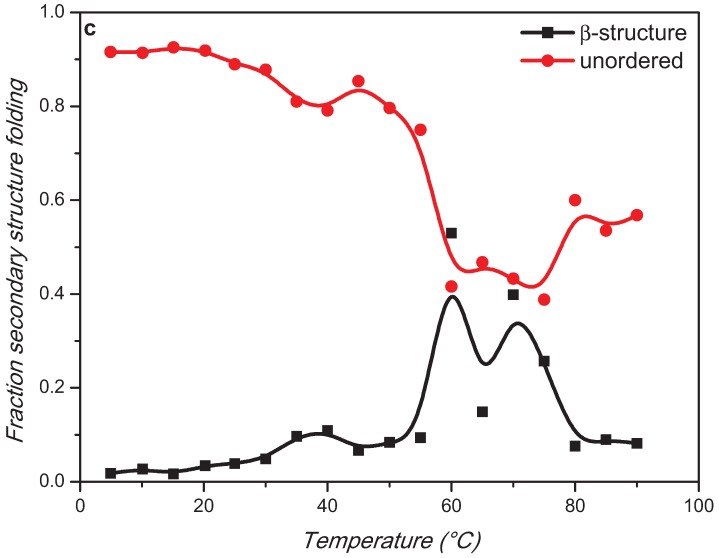
Secondary structure β-strand and unorder components melting curves of α-synuclein alone (**a**) or in presence of 10 mM (**b**) or 100 mM (**c**) trehalose.

The estimation of the protein secondary structure content for α-synuclein and in presence of 10 mM and 100 mM trehalose at different temperatures from the experimental results of Figure 3 was performed using CDApps [25] software containing CONTINLL algorithm (standard deviation between 0.02 and 0.15) [26] (Figure 5).

For α-synuclein, the estimation revealed that the high content of unordered structure remained constant until about 50 °C and then decreased with a corresponding increased content β-sheet structure (Figure 5).

In presence of trehalose, the trends of each element of secondary structure revealed an abrupt change that was not apparent in Figure 3 and Figure 4. The conversion of unordered to β-strand structure in the presence of 10 mM trehalose appeared to peak at 70 °C quickly reversing back to disorder conformation already at 80 °C, which was retained upon cooling back to 20 °C as seen in Figure 3. At 100 mM trehalose, this conversion appeared to start at lower temperature 55 °C with the loss of β-strand conformation at 80 °C. The unordered structure, however, does not recover fully back at 20 °C suggesting other structural components such as β-turns and/or α-helix conformations might be promoted.

Collectively, these data indicated that the thermodynamic property of α-synuclein folding was perturbed by the presence of trehalose in a dose-dependent manner. This trehalose effect is more evident at low concentration showing thermal reversibility.

Interestingly, at the physiological temperature (range 30–35 °C) the content in secondary structure of α-synuclein is not affected by the presence of low concentration of trehalose, while an increase in the amount of β-strand conformation has been detected in the presence of 100 mM trehalose suggesting a predominant exclusion phenomenon [23], which promotes protein compaction.

## 3. Experimental Section

### Synchrotron Radiation Circular Dichroism Spectroscopy

Synchrotron radiation circular dichroism (SRCD) experiments have been performed at the Diamond beamline B23. Trehalose was obtained from Sigma-Aldrich (Milan, Italy) and used without further treatment. α-Synuclein was expressed in *E. coli* BL21 (DE3) strain following the procedure described in Marchiani *et al.* [20] and dissolved in phosphate saline buffer (PBS), pH 7.4, at concentration of 0.36 mg/mL. Concentrated stock solutions of trehalose (1100 and 110 mM, respectively) were prepared in the same buffer. SRCD spectra from 180 to 260 nm were collected at the beamline B23 module end station B, bandwidth = 1.1 nm, integration time of 1 s, 1 nm digital resolution, 39 nm/min scan speed, and one repeated scan per spectrum, using Suprasil cell (Hellma GmbH, Mülheim, Germany) of 0.02 cm pathlength. The thermal stability was monitored in the 5 °C to 90 °C temperature range (increment steps of 5 °C) using a Quantum Peltier temperature controller. Temperature was equilibrated for 5 min before collecting the spectra. SRCD spectra have been analyzed using CDApps software [25].

## 4. Conclusions

Our results suggest that trehalose can interact with α-synuclein, affects its folding property in dose-dependent manner and might inhibit its aggregation process. Whether trehalose prevents fibrillation by either inhibiting protofibrils assembly or by destabilizing protofibrils or both is still a matter of debate. The hypothesis that the early oligomers or protofibrils might be the cytotoxic species responsible for neuronal cell death [27] implies that inhibitors of protein oligomerization could be used successfully for the therapy of neurodegenerative diseases. Our finding is consistent with this view and could have a putative use in the treatment of Parkinson’s disease (PD). In conclusion, we found that low concentration of trehalose stabilizes α-synuclein folding, and blocks *in vitro* polymerization better than high trehalose concentration. As trehalose is widely used as excipient in pharmaceutical formulations, our data suggest that this molecule could be associated with other drugs to develop a promising new approach for treating PD and other brain-related diseases.
